# Circadian Rhythm Regulates Development of Enamel in Mouse Mandibular First Molar

**DOI:** 10.1371/journal.pone.0159946

**Published:** 2016-08-05

**Authors:** Jiang Tao, Yue Zhai, Hyun Park, Junli Han, Jianhui Dong, Ming Xie, Ting Gu, Keidren Lewi, Fang Ji, William Jia

**Affiliations:** 1 Department of General Dentistry, Ninth People's Hospital, Shanghai Jiao Tong University School of Medicine, Shanghai Key Laboratory of Stomatology, Shanghai, China; 2 Department of Prosthodontics, Ninth People's Hospital, Shanghai Jiao Tong University School of Medicine, Shanghai Key Laboratory of Stomatology, Shanghai, China; 3 Department of Oral Pathology, Ninth People's Hospital, Shanghai Jiao Tong University School of Medicine, Shanghai Key Laboratory of Stomatology, Shanghai, China; 4 Department of Medicine, Windsor University School of Medicine, St. Kitts & Nevis; 5 Department of Orthodontics, Ninth People's Hospital, Shanghai Jiao Tong University School of Medicine, Shanghai Key Laboratory of Stomatology, Shanghai, China; 6 Brain Research Centre, Department of Surgery, University of British Columbia, Canada; University of Lübeck, GERMANY

## Abstract

Rhythmic incremental growth lines and the presence of melatonin receptors were discovered in tooth enamel, suggesting possible role of circadian rhythm. We therefore hypothesized that circadian rhythm may regulate enamel formation through melatonin receptors. To test this hypothesis, we examined expression of melatonin receptors (MTs) and amelogenin (AMELX), a maker of enamel formation, during tooth germ development in mouse. Using qRT-PCR and immunocytochemistry, we found that mRNA and protein levels of both MTs and AMELX in normal mandibular first molar tooth germs increased gradually after birth, peaked at 3 or 4 day postnatal, and then decreased. Expression of MTs and AMELX by immunocytochemistry was significantly delayed in neonatal mice raised in all-dark or all-light environment as well as the enamel development. Furthermore, development of tooth enamel was also delayed showing significant immature histology in those animals, especially for newborn mice raised in all daylight condition. Interestingly, disruption in circadian rhythm in pregnant mice also resulted in delayed enamel development in their babies. Treatment with melatonin receptor antagonist 4P-PDOT in pregnant mice caused underexpression of MTs and AMELX associated with long-lasting deficiency in baby enamel tissue. Electromicroscopic evidence demonstrated increased necrosis and poor enamel mineralization in ameloblasts. The above results suggest that circadian rhythm is important for normal enamel development at both pre- and postnatal stages. Melatonin receptors were partly responsible for the regulation.

## Introduction

Melatonin[1] is mainly secreted from the pineal gland during the darkness and acts via G protein-coupled receptors as a hormonal message of the photoperiod[2]. Circadian responses to melatonin are mediated by melatonin receptors. In mammals, there are two melatonin receptor subtypes, termed MT1 and MT2 melatonin receptors. While MT1 and MT2 are mostly distributed in the pars tuberlis (PT) of pituitary gland[3, 4] and the suprachiasmatic nucleus(SCN)[4, 5]in the brain, they have also been found in many tissues, including the retina, liver, lung, skin, choroid plexus, Harderian gland, adrenal cortex and reproductive organs[6–11]. Interestingly, MT1 was reported to express in human teeth and rat dental epithelial cell line HAT-7[12]. Melatonin is known as “the hormone of darkness”[2] with a variety of physiological actions controlling circadian rhythms of the body. The rhythmic controlling is peaked during the night but only minimal during the day [13, 14]. Recent studies have reported that melatonin enhances the differentiation of osteoblasts in vitro and promotes bone formation in vivo [15–19] and changes in endogenous melatonin production or alterations in melatonin receptor expression have also been shown in circadian rhythm sleep disorders, Alzheimer’s and Parkinson’s diseases, etc [20–22].

Circadian rhythms are commonly present among living organisms and the predentin matrix synthesis, the mineralization systems of teeth, morphology of odontoblast cell and tooth eruption are no exception[23, 24]. Tooth development progresses through stages of initiation, bud, cap, early bell and late bell[25]. Teeth arise from sequential and reciprocal interaction between cells that have migrated from the oral epithelium and the cranial neural crest[26]. During tooth development, ameloblasts and odontoblasts are lined up face-to-face or basement membrane-to-membrane differentiate at the dentin-enamel junction (DEJ)[26, 27]. The ameloblasts move outward toward eventual tooth surface and leave a trail of secreted mixture of proteins which undergoes self- assembly to form an enamel extracellular organic matrix and eventually almost completely replaced by inorganic crystallites during maturation[28]. The odontoblasts which differentiate into columnar cells and help to form the mantle dentin move away from the DEJ toward the future pulp [29, 30]. Among the rest dental structure including dentin and cemetum, enamel has been studied in the most details for its important role in odontogenic development [23, 24].

Enamel is secreted by cells known as ameloblasts, which are differentiated at the DEJ and left tracks known as enamel prisms[31]. The prisms show cross-striations, whose formation relates to the ameloblasts’ daily periodicity formation of enamel with an interval of 2–6μm perpendicularly cross prisms [16, 24]. Retzius lines, also known as enamel incremental lines, are preserved as long-period incremental structures, whose intervals represent 5–10 days’ deposits of enamel[32–34]. Perikymata, circumferential rings on the surface of tooth, is another evidence of tooth’s circadian rhythmic development [34, 35].

Unlike other mineralized tissues, mature enamel is the most durable part of the body and has a unique hierarchical structure, composed largely of carbonated hydroxyapatite, Hap, and very few protein remnants[36]. Amelogenin is the dominant protein in the structural organization and biomineralization of enamel, comprise more than 90% of the extracellular matrix proteins in the secretory stage of the enamel formation [31, 37]. Amelogenin was thought to be an enamel protein only. However, recent studies have found amelogenin in the odontoblasts, the dentin matrix and in periodontal ligament cells and etc [38–41]. Reports have shown the expression of amelogenins in long bone cells, cartilage and some nonmineralizing tissues such as the brain and eye, which possibly reflect other functions of amelogenin[42]. Amelogenin knockout transgenic mice appeared with reduced thickness of enamel and hypoplastic amelogenesis imperfecta, which strongly suggest that the amelogenin takes an essential part in enamel formation [43]. Mutations in the X-chromosomal copy of the amelogenin gene AMELX have been associated with the hereditary disease Amelogenesis Imperfecta in human [42, 44].

In human, with the maturation of the synthesis and secretion system of melatonin, the increasing in melatonin level begins in 6-8th week of life, and the well established circadian rhythm is formed in 21-24th week of life. Amplitude of melatonin secretion reaches its peak between 4th and 7th year of age and begins to drop around maturation (12th year of age)[45]. For tooth, the mineralization of the permanent tooth begins in 3-4th month of life, the deciduous teeth start to be replaced by permanent teeth from 6th year of life to 12th year of life. It can be concluded that there is some similarity between the secretion rhythm of melatonin and the development rhythm of tooth. Despite the well-known melatonin’s rhythmic secretion and tooth’s rhythmic growth, the mechanism of the latter, especially the relation to melatonin mediated rhythmic regulation has not been elucidated[46]. As a lipophilic hormone, melatonin can easily cross the placenta barrier. Thus, the melatonin level of mother can affect that of the fetal[47]. In the present study, we have studied the role of circadian rhythm in molar development in neonatal mice. Although MT in many parts of tooth germs and the effects of melatonin on dental papilla cell and dentine formation have been reported [12, 48–50], we are the first to prove that melatonin mediated by day/night circadian rhythm is involved in normal development of enamel at both pre- and postnatal stages. Our results have also shown that melatonin receptors are important players in this rhythmic regulation.

## Materials and Methods

### Animals

BALB/c mice were purchased from Shanghai Animal Experiment Center. All experiments were approved by the Animal Welfare Committee of Shanghai Ninth People’s Hospital School of Medicine, Shanghai Jiao Tong University.

### Control Group

76 neonatal mice whose mother was raised in artificial illuminations with 12 hours light/dark cycle were sacrificed(mice were euthanized with an intraperitoneal injection of 0.05 mg/g sodium pentobarbital) at D-0 (the day of birth), D-1, D-2, D-3, D-4, D-5, D-6, D-7 and D-10.

6 E18.5 and 6 E19.5 pregnant mice were sacrificed and 12 mouse embryos were got separately.

### Non-Circadian Rhythmic Animals

30 E16 pregnant mice were separated randomly into “DAY”, “NIGHT” and “control” groups. Each pregnant mouse was raised separately. DAY groups were raised in a room with 24 hours illumination (40 watts light bulb positioned 1m away), “NIGHT” groups were raised in complete darkness and the “control” animals were raised in regular light/dark 12/12 hrs cycle. Two newborn mice were sacrificed immediately after birth in “NIGHT” groups and “DAY” groups separately.

Other newborn mice were continuously raised with their mothers in the above conditions. Two baby mice in every “NIGHT” groups and “DAY” groups were sacrificed on D3. After D3, all the mice in “NIGHT” groups and “DAY” groups were raised in regular light/dark 12/12 hrs cycle and two baby mice in the previously “NIGHT” groups and “DAY” groups were sacrificed on D4. Baby mice not born in the expected date were removed from their group without further experiments.

### Tissue Preparation and Immunohistochemistry

The skull of neonatal mice of all groups were split along the sagittal midline, fixed by immersion in 10% neutral formaldehyde for 24 hrs, and processed routinely for paraffin embedding. Sections contained mandibular first molar were cut into 4μm sections, deparaffinized, treated by citric acid(0.01mol/L, pH 6.0) for heat-induced epitope retrieval 20 minutes at 100°C and immunostained with MEL-1A/B-R(H-120) antibody (Santa Cruz Biotechnology, USA) at a concentration of 1:50 in PBS (PH 7.4, 4°C) and AMELX antibody (Abcam, USA) at a concentration of 1:200 in PBS (PH 7.4, 4°C) overnight. All sections were then incubated with a secondary antibody using EnVision (HRP RABBIT/MOUSE) (Dako, Denmark) at a concentration of 1:150 in PBS (Ph 7.4) room temperature for 30 minutes. The immune staining was visualized with 3,3’-diaminobenzidine (DAB), and counterstained with hematoxylin.

### Treating the Mice with 4P-PDOT

4-phenyl-2- propionamidotetraline(4P-PDOT) is a MT2-selective antagonist. 30 E16 pregnant mice were treated with intraperitoneal injection of 4P-PDOT(Tocris, Bristol, UK). The injection dose is 25ug/100g.

### Measuring the Height of Ameloblasts and Image-Pro Plus

Tooth samples of 3-day-old neonatal mice were randomly picked from three different litters of control groups and DAY groups, respectively. Each sample was cut into three sections and stained with hematoxylin and eosin. After taking pictures of ameloblasts of mandibular first molar from those sections at X400 magnification in an optical microscope, heights of the ameloblast layer were measured in three random locations using Image-Pro Plus software by a person blinded to the treatment. The heights of the ameloblast layer were calculated as the average of the three measurements.

### Dissection of Tooth Germs and Real-Time PCR

Each side of the mandibular first molar tooth germs from all groups were dissected under microscope. After dissection, total RNA were extracted using TRI-REAGENT standard protocol. Reverse transcription were carried out by MMLV with oligo (dT) as primer. Semi-quantitative Real-Time PCR was carried out by Bestar SybrGreen qPCR Mastermix (DBI, Germany) to test related gene temporal expression pattern. PCR amplification was performed using cDNA as the template and the conditions were 95°C for 2 min, 60 and72°C 30s, followed(F) and reverse (R) primers for MT1, MT2, AMELX1, AMELX2 were used for Real-Time PCR (Table 1).

**Table 1:** 

Gene names	Primer sequences, forward (F) /reverse (R)
MT1	(F): 5'-TGCCCAACCTGCAAACCGGA-3'
	(R): 5'-TGGCAGGGTCTGAGGCCACA-3'
MT2	(F): 5'-ACACTCACTGGGCGGGGAGG-3'
	(R): 5'-GGGCCTCCCCAAGGACCCAA-3'
AMELX1	(F): 5'-TTTGTTTGCCTGCCTCCT-3'
	(R): 5'-GCTGATGGTGTTGGGTTG-3'
AMELX2	(F): 5'-CCTGGAAGCCCTGGTTAT-3'
	(R): 5'-GGATGGAGGGATGTTTGG-3'
β-ACTIN	(F): 5'-GGAGATGGCCACTGCCGCAT-3'
	(R): 5'-GCAGCTCAGTAACAGTCCGCCTA-3'

### Dissection of Tooth Germs and Electron Microscopy

At least six mandibular first molar tooth germs from 7-day-old mice of both control and PDOT-treated groups were dissected, fixed in 2% glutaraldehyde phosphoric acid buffer for 24 hrs (4oC), decalcified in 4% EDTA-2Na solution for 24 hrs, and processed for transmission electron microscope (TEM) (PHILIP CM-120 TEM).

### Statistical Analysis

The results were expressed as the means ± S.D. The statistical tests used to assess the difference were determined by Student’s t-test. Statistical significance was set at a P value <0.05.

## Results

### Expression of MTs and Amelogenins in the Developing Enamel

#### mRNA expression

**Fig 1:**
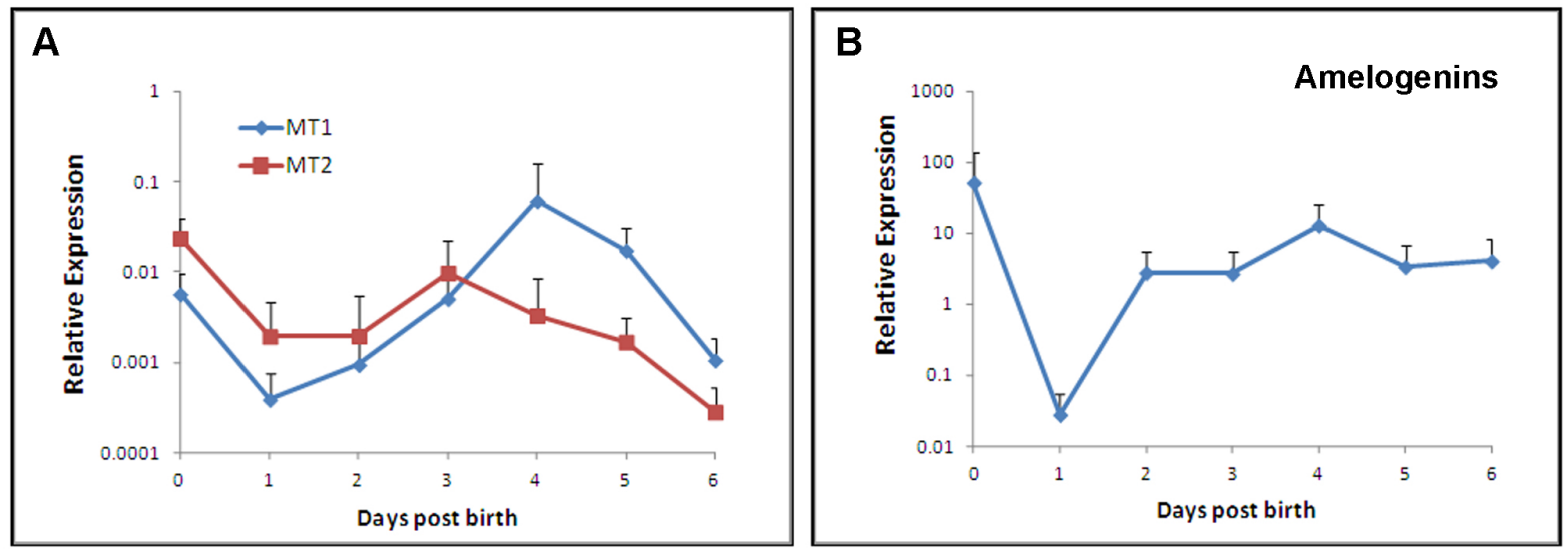
Expression of MT1, MT2 (A) and amelogenins (B) by qRT-PCR in developing enamel of the mandibular first molar of mouse at different ages. The results of the qRT-PCR are depicted as means ± SD (n = 30 in each group) * p<0.05, **p<0.01.

Quantitative RT-PCR was performed to measure the transcripts of MT1 and MT2 as well as amelogenins in developing enamel of the mandibular first molar of mouse. As shown in Fig 1A, levels of MT1 and MT2 mRNAs were relatively high at D0 but significantly reduced at D1. The expression of MT1 mRNAs increased with age and peaked around D4. Levels of both MT1 and MT2 transcripts declined by the end of first week after birth. It seems that MT1 mRNA is dominant at D4. Amelogenins mRNA was also high at birth and dramatically declined by more than 100-fold at D1 (Fig 1B). Unlike MTs, levels of amelogenins transcripts rapidly elevated by D2 and remained that level till D6 S1 File.

#### MT protein distribution

**Fig 2:**
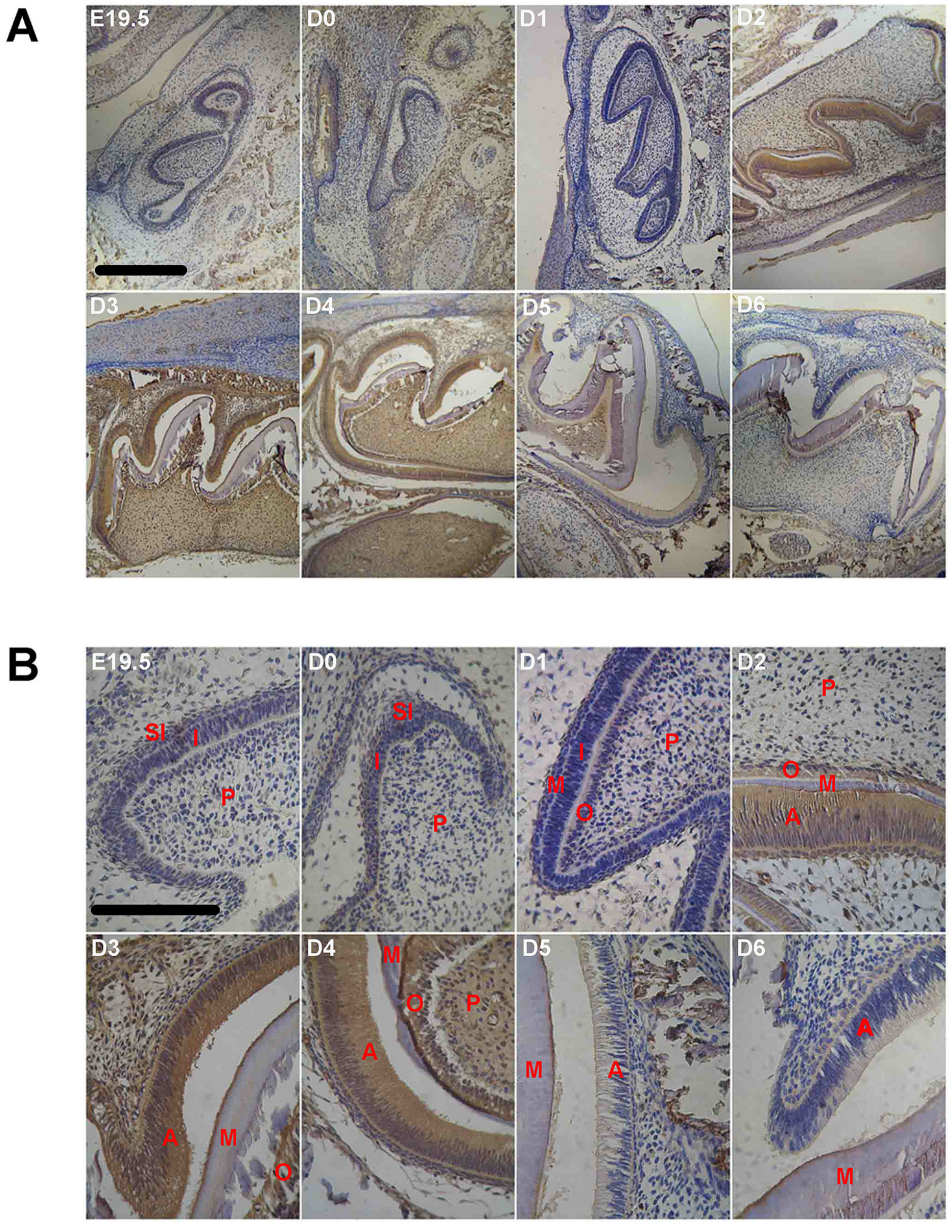
Immunocytochemistry of melatonin receptors in developing first molar tooth germ of mice at different ages (n = 6 in each age group). A, microphotograph with 10x objective; B, microphotograph with 40x objective. Scale bar, 10μm. Labels in photos: A, ameloblast; AB, alveolar bone; I, inner enamel epithelium; M, mineralization layer; O, odontoblast; P, dental papilla cell; SI, stratum intermedium.

We have also examined expression of MTs at the protein level with immunocytochemistry. Since the antibody utilized in the present study did not distinguish MT1 and MT2, results of the immunocytochemistry staining was therefore for total MT proteins. Nevertheless, expression of MT proteins in developing enamel generally agrees with their message RNAs. Fig 2A and 2B show the MT immunoreactivity in the dental organ at various ages. At postnatal D0, the day the mice were born, the tooth germ was still in late cap stage, when the inner enamel epithelium cells appeared in monolayer and low columnar. A weak but definitive MT positive staining was evident in the inner enamel epithelium cells, the stellate reticulum cells, alveolar bone and mandibular. Interestingly, while the enamel organ had further differentiated at D1 into early bell stage with clearly differentiated columnar inner enamel epithelium cells and polarized, high columnar odontoblasts, the immune staining for MTs was barely detectable, which was quite consistent with the expression of MT mRNAs. Strong staining was again shown in ameloblasts of D2-D4 mice (Fig 2B) whose enamel organs were at the late bell stage (Fig 2A). In addition, MT immune-positivity was also seen in odontoblasts, dental papilla cells, alveolar bone and mandibular (Fig 2B). In consistence with the developmental profile of the MT mRNAs, the immune reactivity was reduced in animals of late ages after postnatal D4.

#### Amelogenins protein

**Fig 3:**
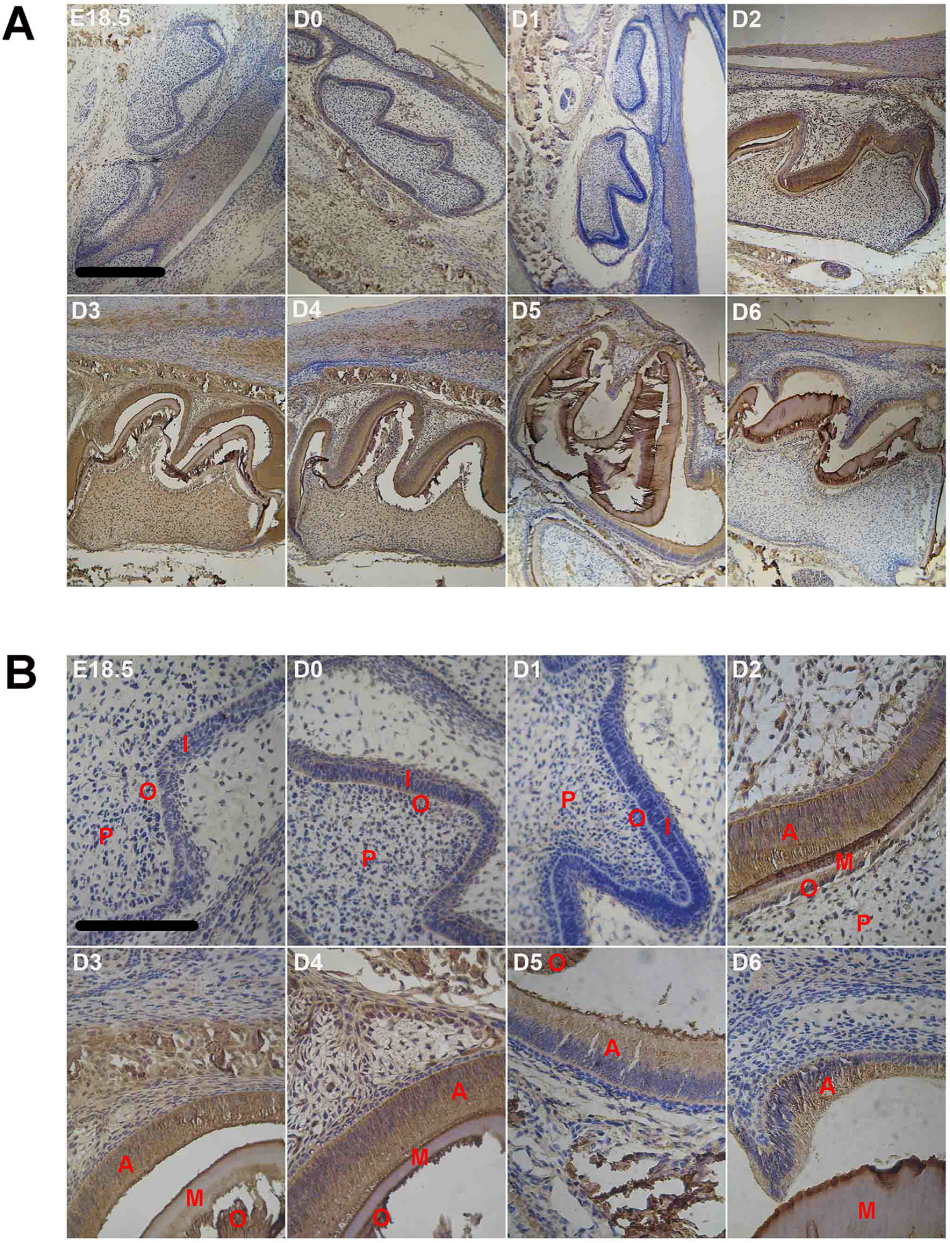
Immunocytochemistry of amelogenins proteins in developing first molar tooth germ of mice at different ages (n = 6 in each age group). A, microphotograph with 10x objective; B, microphotograph with 40x objective. Scale bar, 10μm. Labels in photos: A, ameloblast; I, Inner enamel epithelium; M, mineralization layer; O, odontoblast; P, dental papilla cell; SI, stratum intermedium.

Similarly, immune staining for amelogenins was clearly evident at D0 (Fig 3D0) but dramatically reduced to undetectable levels at D1 in the enamel organ (Fig 3D1). Starting from D2, the amelogenins staining became very strong in ameloblasts, odontoblasts and mineralizing matrix between them (Fig 3B). Amelogenins immune-positivity was also seen in dental papilla cells, alveolar bone and mandibular, the positions of which were quite the same as where MT protein.

The staining intensity remained high with a slight reduction after postnatal D5 (Fig 3B, 3D5 and 3D6).

### Expression of MTs and Amelogenins Is Regulated by Circadian Rhythm

**Fig 4:**
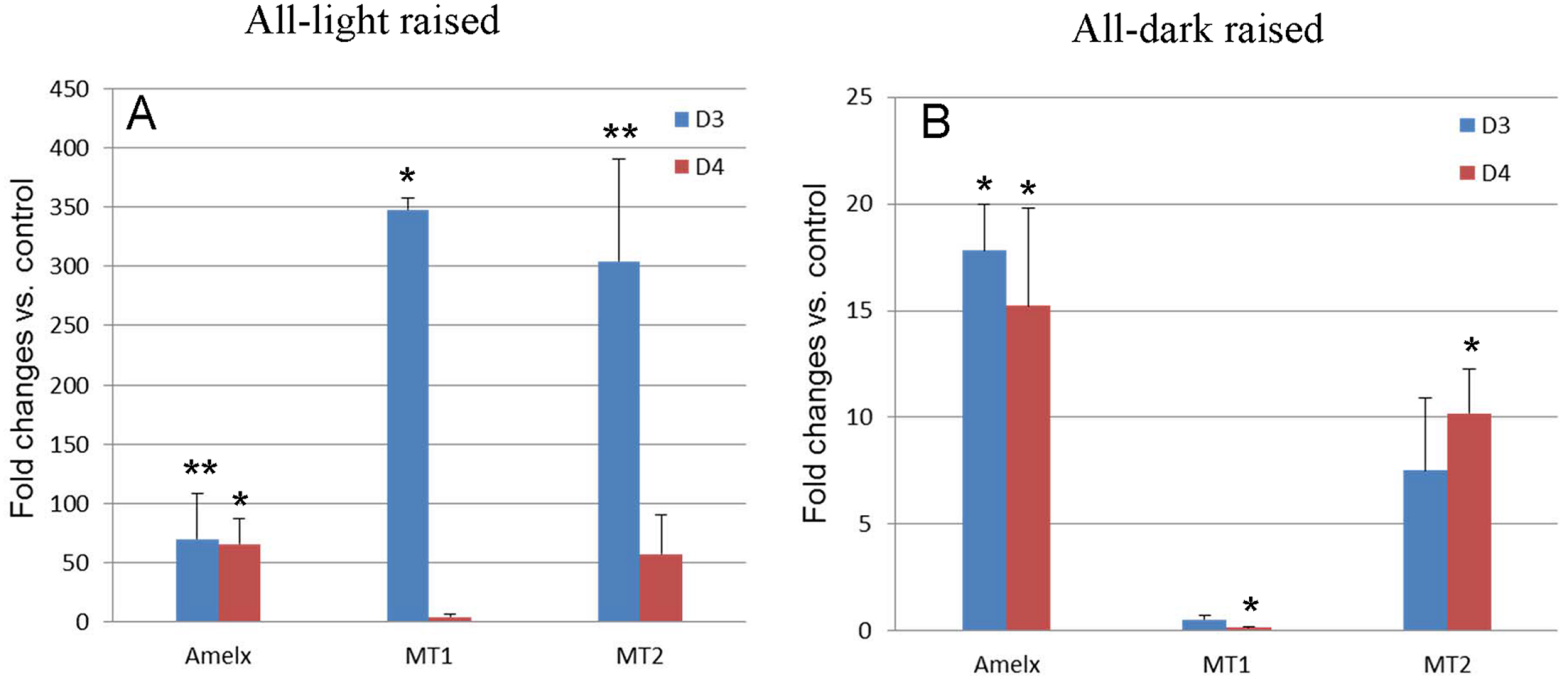
Messenger RNA by qRT-PCR of MT1, MT2 and amelogenins in developing enamel of the mandibular first molar in mice experienced all dark or all light since birth. D3, animals deprived daylight or darkness for 3 days since birth. D4, animals deprived daylight or darkness for 3 days followed by one day of normal day-night cycle. The results of the qRT-PCR are depicted as means ± SD (n = 3 in each group).*, p<0.05, **, p<0.01.

To answer the above question, we raised neonatal mice in either a completely lighted or completely dark environment since they were born till postnatal day 3 followed by one cycle of day-night. Some animals were killed by the end of D3 (no circadian experience) and the rest were killed by the end of D4 (with one day-night cycle). Fig 4 shows changes in levels of amelogenins and MTs mRNA in postnatal tooth germs at day 3 and day 4. Deprivation of night (i.e. all-light raised) for 3 days postnatally resulted in a dramatic transcriptional upregulation of both amelogenins and MTs by ~70-fold and ~350-fold in the tooth germs, respectively (Fig 4A), comparing to the control group of the same age. Upregulation of transcripts for amelogenins and MT2 but not MT1 were also seen in all-dark raised newborn mice at day 3 although to a less extent (Fig 4B).

MT mRNAs in mice raised in all-light environment for 3 days since birth showed dramatic reduction after one cycle of day-night but amelogenins mRNAs remained high(Fig 4A). However, the first cycle of circadian rhythm had no effect on transcription of amelogenins and MT2 in all-dark raised mice (Fig 4B)S2 File.

**Fig 5:**
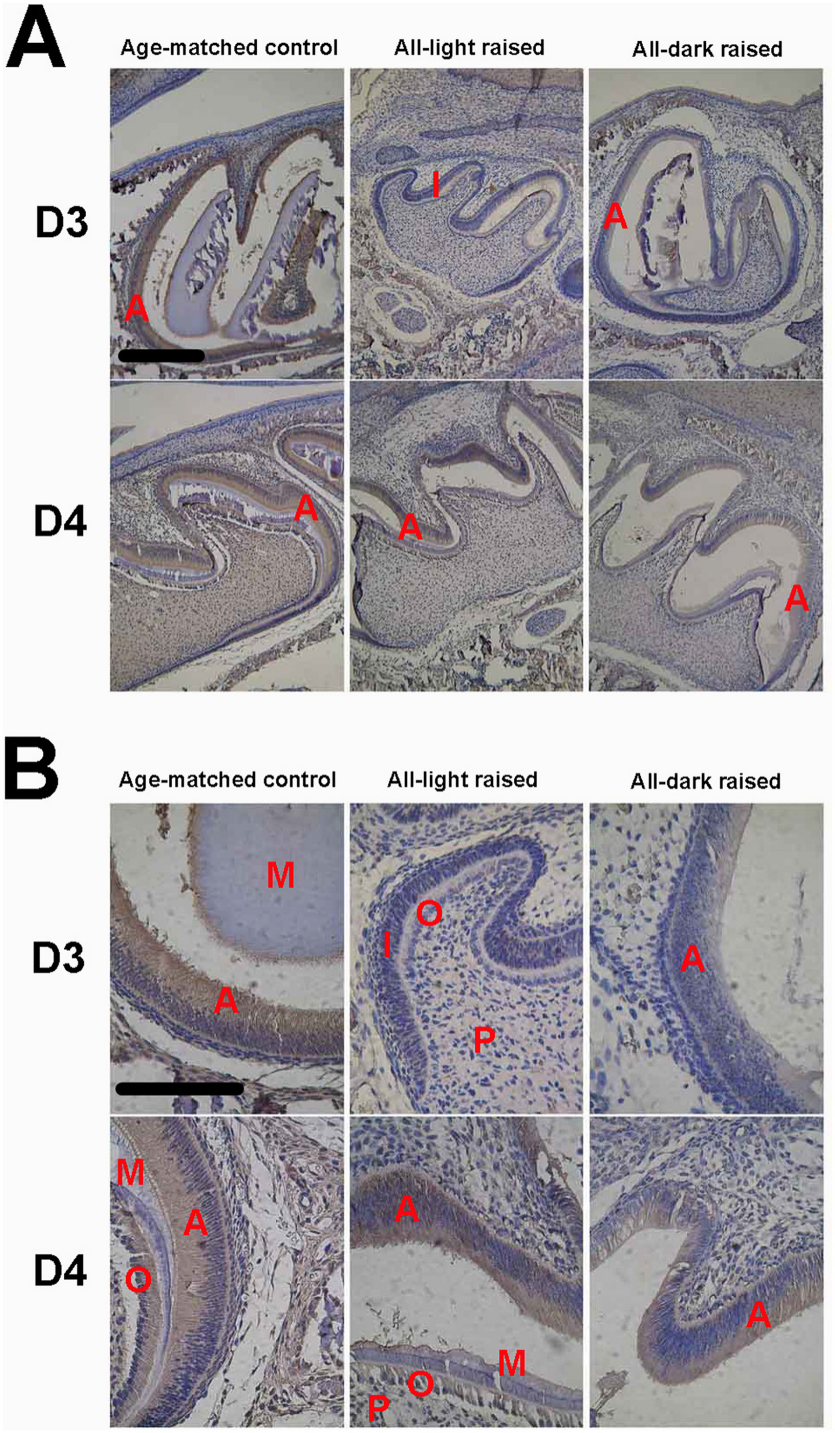
Immunocytochemistry of melatonin receptors in the first molar tooth germ of mice raised in either night-deprived or daylight-deprived conditions (D3) as well as mice experienced three day circadian rhythm derivation followed by one day-night cycle (D4) (n = 6 in each group). A, microphotograph with 10x objective; B, microphotograph with 40× objective. Scale bar, 10μm. Labels in photos: A, ameloblast; I, inner enamel epithelium; M, mineralization layer; O, odontoblast; P, dental papilla cell.

**Fig 6:**
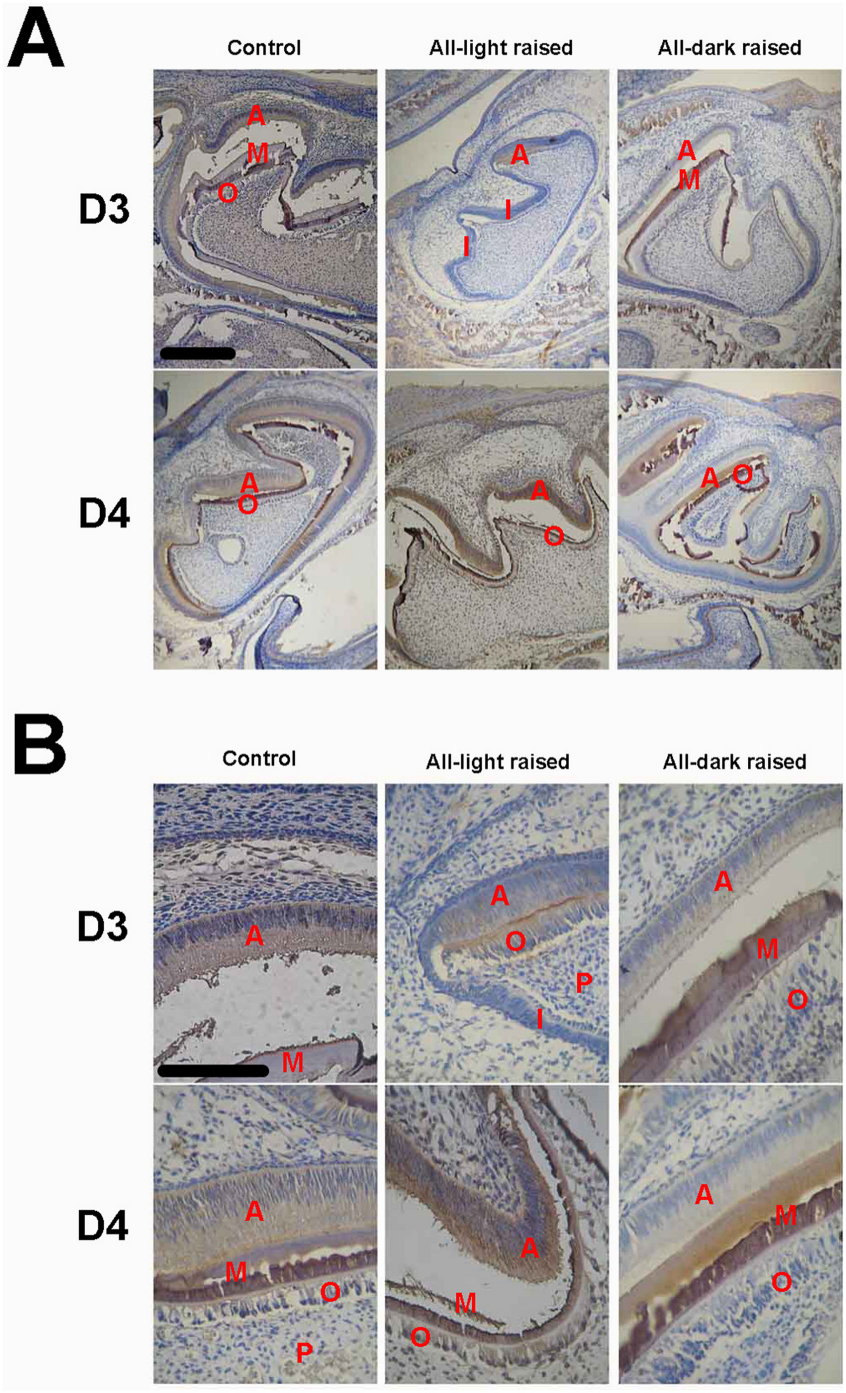
Immunocytochemistry of amelogenins in the first molar tooth germ of mice raised in either night-deprived or daylight-deprived conditions (D3) as well as mice experienced three day circadian rhythm derivation followed by one day-night cycle (D4) (n = 6 in each group). A, microphotograph with 10x objective; B, microphotograph with 40x objective. Scale bar, 10μm. Labels in photos: A, ameloblast; I, inner enamel epithelium; M, mineralization layer; P, dental papilla cell; O, odontoblast.

We also compared the protein levels of MTs and amelogenins by immunocytochemistry in the enamel organs of postnatal D3 and D4 mice (Figs 5 and 6). In contrast to their mRNAs, both MTs and amelogenins proteins were underexpressed in the tooth germs in D3 mice raised in a non-circadian environment compared with age-matched control animals.

The development of enamel organs and the differentiation of ameloblasts were also remarkably delayed in those animals, especially in all-light raised mice. As shown in Figs 5A and 6A, the enamel organ was in late bell stage in postnatal day 3 of normal mice, when ameloblasts and odontoblasts were in secretary stage and the mineralizing matrix between them could be observed clearly. In contrast, differentiation of the tooth germs was significantly delayed in animals raised in non-circadian environment, especially for all-light raised animals (Figs 5 and 6), showing ameloblasts differentiated only in cuspids of molar tooth germs and the rest parts were still inner enamel epithelium cells, which have not been differentiated into ameloblasts yet and the tooth germs were relatively small. Since the cuspids is the earliest structure for mineralization of teeth 40, these results further confirmed that the development of tooth germs in all-light raised animals are indeed delayed. Although the tooth germs of all-dark raised animals on postnatal day 3 showed differentiated ameloblasts, obvious mineralized matrix could not be observed, which also demonstrated its delayed development. Interestingly, once the animals were exposed to a day-night cycle (D4), not only the levels of MTs and amelogenins proteins were dramatically increased to near the level of that of animals raised in the normal environment, the tooth germs were also rapidly differentiated into the morphology similar to their age-matched counterparts. After one circadian cycle, all-light raised mice showed significantly bigger size of mandibular first molars, whose ameloblasts were in secretary stage and moved oppositely to odontoblasts which enlarged the space between them, but the mineralizing matrix was not obvious. Compared with all-light raised mice, the daylight-deprived mice at D4 showed obvious mineralizing matrix that is similar to the control mice.

### Circadian Rhythm and Melatonin Receptors in Pregnant Mice Affect Development of the Tooth Germs in the Newborns

**Fig 7:**
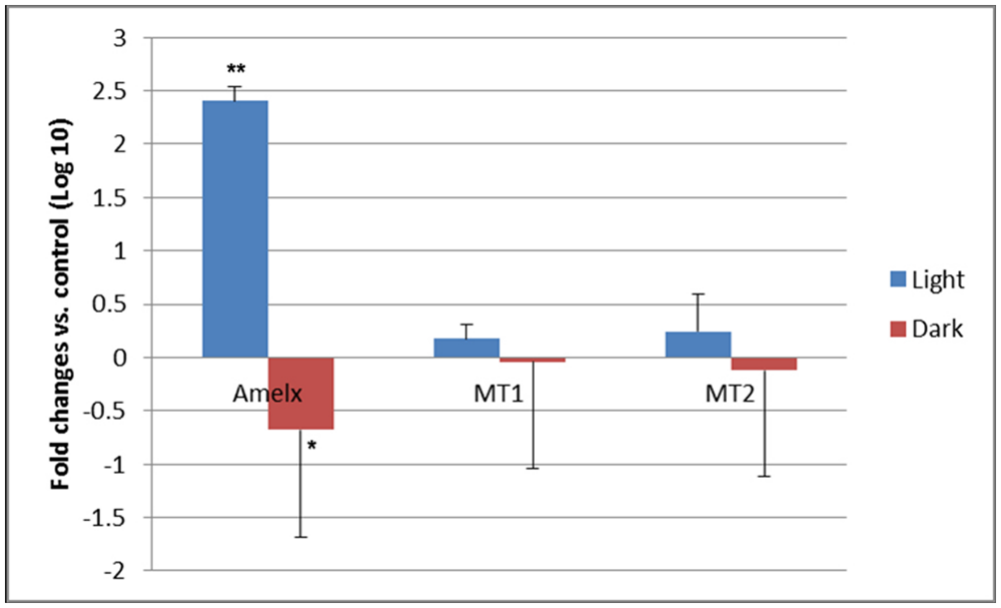
Expression of MT1, MT2 and amelogenins by qRT-PCR in developing enamel of the mandibular first molar of newborn mice from mothers with deprived circadian rhythm. The results of the qRT-PCR are depicted as means ± SD (n = 8 in each group). * p<0.05, ** p<0.01.

Next, we asked whether disturbance of circadian rhythm in mothers has any impact on enamel development in the neonatal mice. Pregnant mice were either raised in all- lighted condition since E16 till giving birth or injected with 4P-PDOT to block melatonin receptors. Messenger RNAs for amelogenin and MTs were measured in newborn mice at day 0 immediately after birth. As shown in Fig 7, mRNAs for MTs did not show any significant change in neonatal tooth germ. However, compared to control newborn mice, levels of amelogenin mRNA were ~250-fold higher in those from night-deprived mothers and 50% less in those from mothers kept in dark before giving birth S3 File.

**Fig 8:**
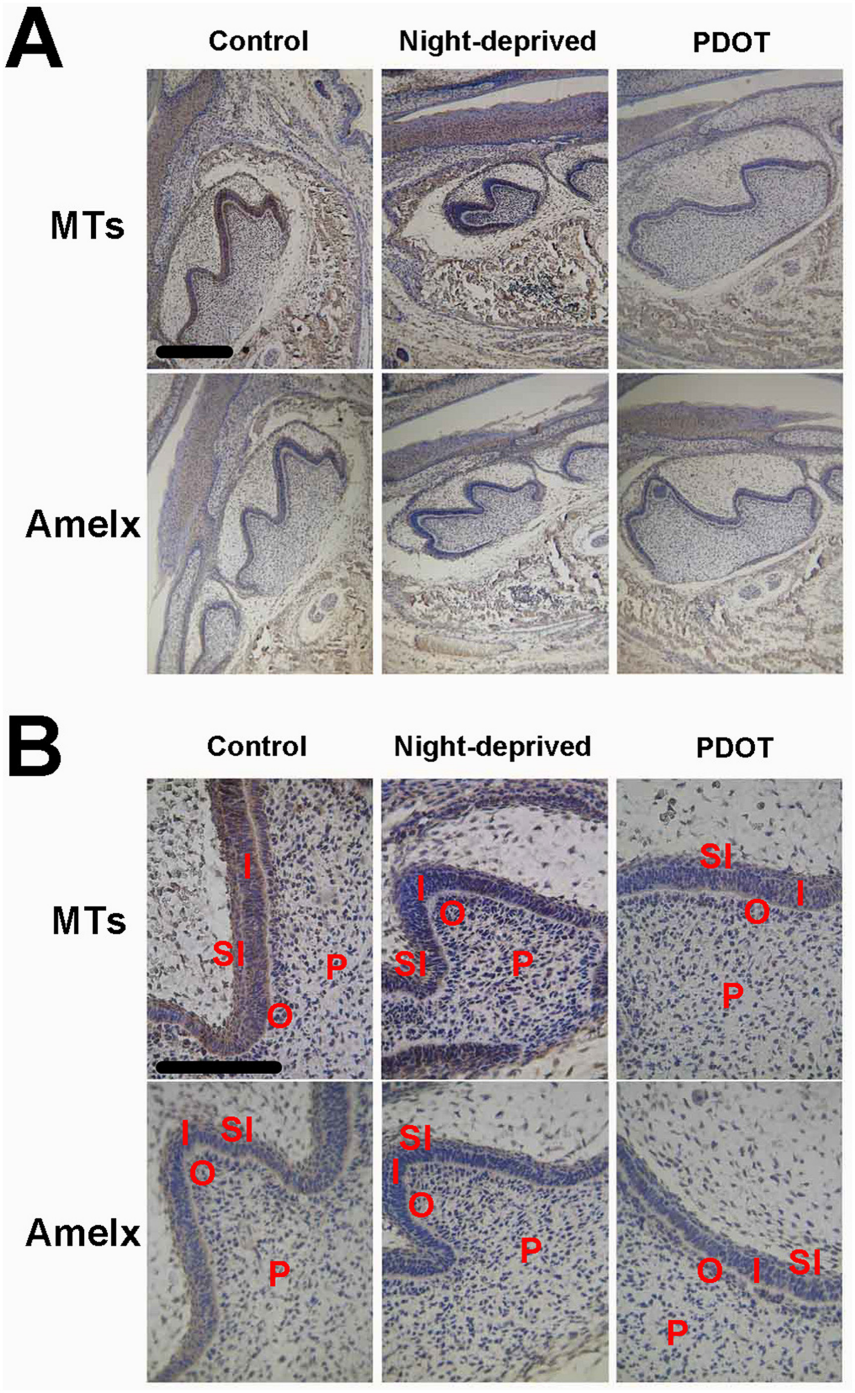
Immunocytochemistry of amelogenin and MT in the tooth germ of newborn mice (D0) from mothers night-deprived or melatonin receptor blocked. A, microphotograph with 10x objective; B, microphotograph with 40x objective. Scale bar, 10μm. Labels in photos: I, inner enamel epithelium; O, odontoblast; P, dental papilla cell; SI, stratum intermedium.

Expression of amelogenins and melatonin receptors were examined in tooth germs of D0 newborn mice from the night-deprived mothers (Fig 8). The enamel organs of postnatal D0 mice in control group were in early bell stage whose inner enamel epithelium has already differentiated into ameloblasts, which shown columnar cells and the odontoblasts opposite to whom shown polarity. However, the enamel organs of postnatal D0 mice from the night-deprived mothers were still in cap stage whose inner enamel epithelium shown single short columnar cells and the odontoblasts opposite to whom hadn’t shown polarity. Furthermore, the protein levels of MTs in those structures are much lower in mice from night-deprived mothers. Protein levels of AMELX in D0 mice of both night-deprived and PDOT-treated groups were also low.

**Fig 9:**
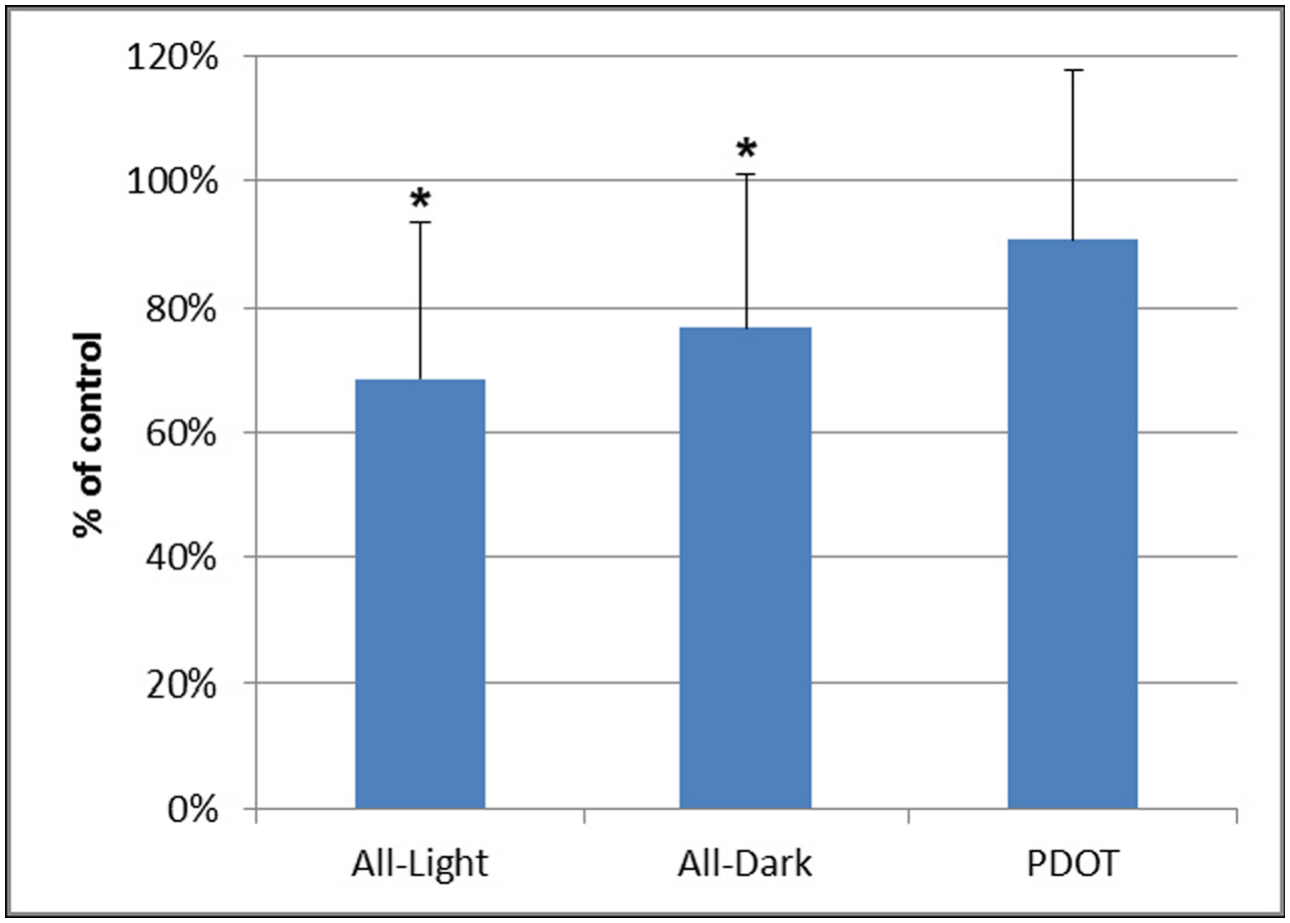
Average heights of ameloblasts (% of control animals) of postnatal day 3 mice of all-day raised, all-night raised and born from 4P-PDOT treated mothers. The data are depicted as means ± SD (9 sections from 3 animals for each group).* p<0.05.

Unlike newborns deprived with night or day for three day after birth, the mice born from 4P-PDOT treated mothers appeared normal enamel morphology at light microscopic level. We measured average height of ameloblasts in tooth germ of postnatal day 3 mice of normal control, all-day raised, all-night raised and born from PDOT treated mothers. As shown in Fig 9, while both all-day and all-night raised groups showed lower height of the ameloblasts, PDOT treatment in the mothers did not significantly affect the height of ameloblasts of their babies.

**Fig 10:**
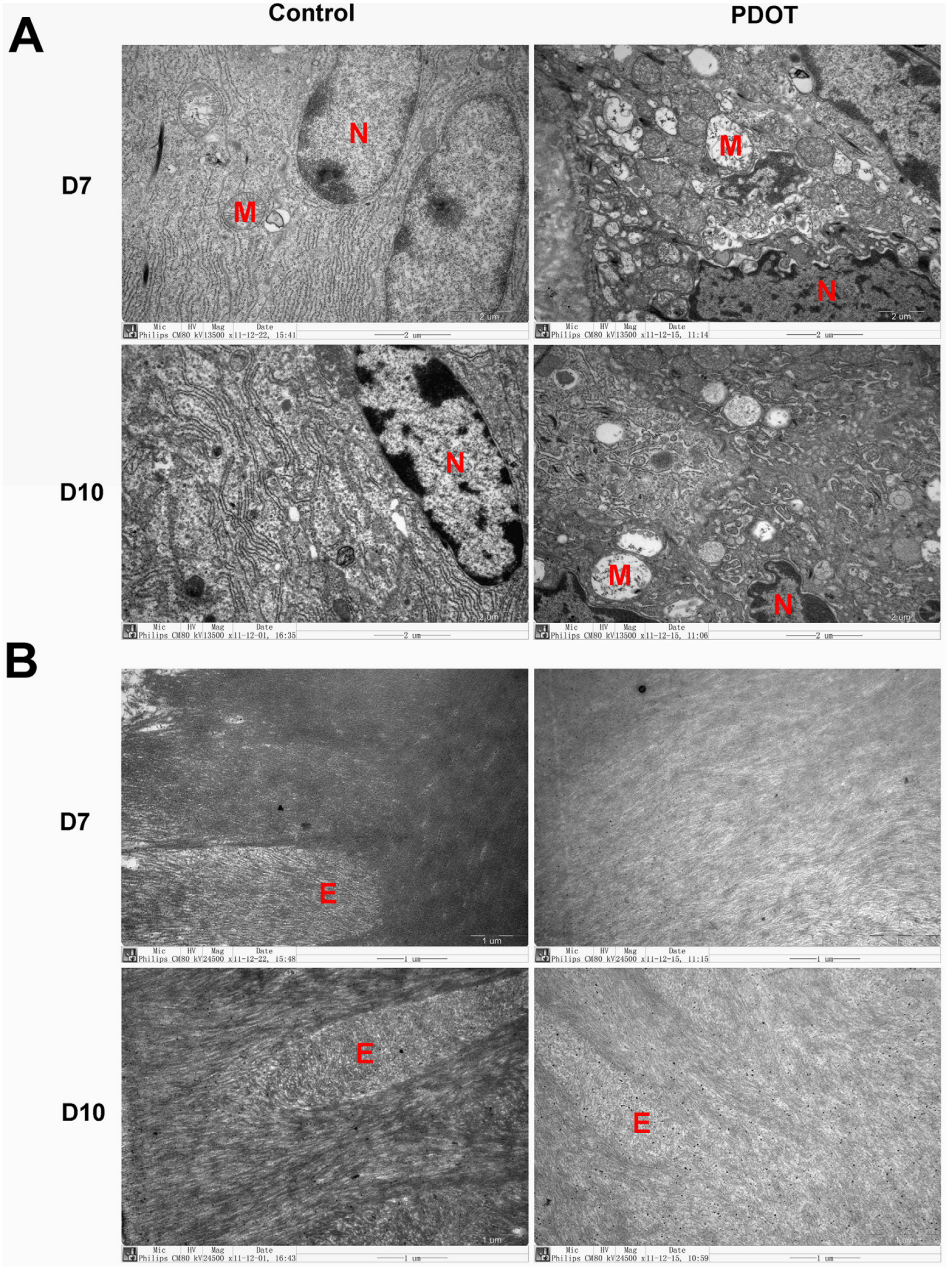
Electromicroscopic photographs of ameloblasts of mandibular first molar tooth germ in baby mice of postnatal day 7 and 10 from mothers treated with 4P- PDOT, a melatonin receptor blocker. A, ultrastructure of ameloblasts, 13500x; B, hydroxyapatite matrix and enamel rod, 24500x. Labels in photos: E, enamel rod; M, mitochondria; N, nucleus. Arrows point to rough surfaced endoplasmic reticulum.

However, electron microscopy revealed profound changes in ameloblasts of mice born by PDOT treated mothers at ultrastructural level (Fig 10). At postnatal day 7, vacuolar degeneration was evident in many ameloblast cells of 4P-PDOT treated animals with extensive pathological dilatation of the rough-surfaced endoplasmic reticulum(RER), reduced number of normal organelles, and disappearance of mitochondrial crista, together with vacuolar degeneration. The degenerating ameloblasts were also often associated with nuclear condensation, as shown in Fig 9A. In addition, abnormal calcification of the enamel was evident in mice from 4P-PDOT treated mothers at both postnatal day 7 and day 10 (Fig 10B). In those baby mice, the edge of enamel rods in the mineralization layer appeared blurry or even unrecognizable; hydroxyapatite crystals were disorganized and finer. Overall, the density of mineralizing enamel was significantly reduced. It is not known whether the long lasting effects of 4P-PDOT on enamel development of newborn mice was the results of residual 4P-PDOT in the newborns’ system or was the irreversible consequence of one time exposure of the prenatal tooth germs to the MT antagonist.

## Discussion

The present study is the first to investigate effect of melatonin mediated by circadian rhythm on enamel development, especially the influence of light and circadian rhythm on expression of melatonin receptors and amelogenins in tooth germs. Our results show that mRNAs of both melatonin receptors and amelogenins dramatically dropped at day 1 after birth. Similar drop in melatonin receptor mRNAs were also seen in new born mice raised in an all-daylight environment for the first 3 days after birth followed by one night cycle. Animals never had experience of circadian cycle since birth showed significantly delayed maturation of tooth germs as well as low protein levels of melatonin receptors and amelogenins. Furthermore, disrupted circadian rhythm of pregnant mother may also affect the enamel development of baby mice as deprivation of night in pregnant mothers caused immature structures of tooth germs and low proteins levels of melatonin receptors and amelogenins in newborn baby teeth. Interestingly, pregnant mice treated with 4P-PDOT, a melatonin receptor blocker, showed same reduction in expression of those proteins. Furthermore, although the tooth germ of newborn mice from 4P-PDOT treated mothers appeared normal, the ameloblasts showed significant vacuolar degeneration associated with reduced mineralization at postnatal day 7 and 10 by electromicroscopic examination.

While MTs are known for their presence in various organs including retina, liver, and gastrointestinal tract during development, this is the first time their expression in the developing tooth germs was studied. Developmental profiles of ameloginins in the tooth organ have been studied in various animals as well as humans [51–53]. Amelogenin mRNA expression was characterized in tooth germs in mouse by in-situ hybridization [51]. However, the results of in-situ hybridization are not comparable with the present study since the former was not for quantitative measurement of mRNAs. A common feature of amelogenin expression among all species is that the primary mRNA transcript undergoes extensive alternative splicing, resulting in several long- and short-spliced mRNA isoforms. In mouse molars, it has been shown that majority of ameloginin transcripts are a long mRNA (M180) that contains exon 6 but not exon 4 while a less abundant full-length mRNA isoform (M194) contains exon 4[51–53]. During the development of mouse molars, expression of amelogenin mRNA transcripts occurs in a restricted time frame [51]. The present study did not separate different mRNA species as the primers used for qRT-PCR recognize all amelogenin mRNAs.

It remains to be elucidated why there is a significant sudden decline of MTs and ameloginins at both transcriptional and translational levels in the tooth germs at postnatal day 1 compared with day 0. One possible explanation is that the transcription of both MTs and amelogenin require certain factors, which are maternally supplied before birth. The transiently reduced expression of MT and amelogenins immediately after birth may reflect the temporal loss of those factors in the newborn mice, which are quickly restored a day after. Alternatively, a possible factor is the first cycle of circadian rhythm that the neonatal mice experienced between D0 and D1. Therefore, we investigated whether or not the day-night cycle attributed to the molecular and histological changes in developing enamel.

Our results clearly demonstrated that circadian rhythm plays crucial role in enamel development as abolishing circadian cycle in newborn mice resulted in significant disturbance in gene expression and delayed development in the tooth germs. In general, either daylight- or night- deprivation caused upregulation in mRNAs of both amelogenins and MTs but downregulation in their proteins. Interestingly, in both cases, effects of the night-deprivation are much more profound than that of daylight deprivation. This may be partially explained by normal behavior of rodents as nocturnal animals that they may be more sensitive to deprivation of night than daylight. It remains to be elucidated for the paradoxical effects of circadian rhythm on transcription and translation of amelogenins and MTs expression. There might be more than one possible explanations. Firstly, synthesis of mRNAs and the proteins of both MTs and amelogenins may be differentially regulated by the circadian rhythm. Secondly, synthesis of corresponding proteins may be blocked due to the disturbed circadian rhythm, leading to the accumulation of corresponding mRNAs. Lastly, the corresponding proteins may have the negative feedback effect towards the synthesis of mRNAs. This feedback mechanism was disrupted due to lack of sufficient proteins after the disturbance of circadian rhythm. More strikingly, deprivation of darkness caused significant delay in enamel development in newborn mice and one circadian cycle drastically restored the process of maturation. All our above results indicate that the circadian rhythm plays a crucial role in regulating development of the tooth germs but detailed study at molecular level in normal development of the tooth germs under physiological conditions is necessary to confirm the above observations.

We also showed that circadian rhythm deprivation in pregnant mice can result in low level of amelogenins at birth and delayed enamel maturation in newborn mice, suggesting that disturbed circadian rhythm in pregnant mother can cause abnormal enamel development of the babies. In addition, the abnormality in enamel development of newborn mice from night-deprived mothers is similar to that from 4P-PDOT treated pregnant mice, suggesting that the effects of circadian rhythm on expression of MTs and amelogenin might be mediated by the MT receptors. However, the abnormality caused by night-deprivation was not totally overlap with that caused by 4P-PDOT, indicating that factors affected by circadian rhythm other than melatonin pathway (such as stress) may also play roles in enamel development. In addition, 4P- PDOT treatment in pregnant mice caused morphologically appeared normal but significantly degenerated ameloblast cells in neonatal mouse teeth further supported the unique functions of MT in regulating differentiation of enamel and tooth calcification.

The current study showed the importance of melatonin mediated by circadian rhythm in regulating enamel development of neonatal animals, which may partially mediated by melatonin signals. Although the pleiotropy of melatonin at the signal transduction pathways has not been sufficiently described [54, 55], parallel signaling via different G protein subunits has been demonstrated for melatonin receptors[56]. MT1 and MT2 mediate intracellular effects which comprise changes in intracellular cyclic nucliotides (cAMP, cGMP) and calcium levels [57–59]. Animal studies shown MT2 activation not only can cause a decline in cAMP, but also an increase level of PKC (protein kinase C)[20, 60], which were specifically blocked by 4-phenyl-2- propionamidotetraline(4P-PDOT), a MT2-selective antagonist[61, 62]. Whether similar routes via MT1 play a role or not remains to be shown and MT2-dependent PKC activation was required for the phase shift of the circadian rhythm[21]. While clinical significance of those results is to be investigated, our findings that abnormal activity of melatonin receptors or loss of circadian rhythm in pregnant mother can significantly delay enamel development of baby mice may have important clinical implication in preventing dental defects of children.

## Conclusion

we found that mRNA and protein levels of both MTs and AMELX in normal mandibular first molar tooth germs increased gradually after birth, peaked at 3 or 4 day postnatal, and then decreased. Expression of MTs and AMELX by immunocytochemistry was significantly delayed in neonatal mice raised in all-dark or all-light environment as well as the enamel development. Furthermore, development of tooth enamel was also delayed showing significant immature histology in those animals, especially for newborn mice raised in all daylight condition. Interestingly, disruption in circadian rhythm in pregnant mice also resulted in delayed enamel development in their babies. Treatment with melatonin receptor antagonist 4P-PDOT in pregnant mice caused underexpression of MTs and AMELX associated with long-lasting deficiency in baby enamel tissue. Electromicroscopic evidence demonstrated increased necrosis and poor enamel mineralization in ameloblasts.

## Supporting Information

S1 File Expression of MT1, MT2 (A) and amelogenins (B) by qRT-PCR in developing enamel of the mandibular first molar of mouse at different ages. (XLSX)

S2 File Messenger RNA by qRT-PCR of MT1, MT2 and amelogenins in developing enamel of the mandibular first molar in mice experienced all dark or all light since birth. (XLSX)

S3 File Expression of MT1, MT2 and amelogenins by qRT-PCR in developing enamel of the mandibular first molar of newborn mice from mothers with deprived circadian rhythm. (XLS)
